# Changes in Volatile Compounds in Short-Term High CO_2_-Treated ‘Seolhyang’ Strawberry (*Fragaria × ananassa*) Fruit during Cold Storage

**DOI:** 10.3390/molecules27196599

**Published:** 2022-10-05

**Authors:** Inhwan Kim, Donghee Ahn, Jeong Hee Choi, Jeong-Ho Lim, Gyeongsik Ok, Kee-Jai Park, Jihyun Lee

**Affiliations:** 1 Department of Food Science and Technology, Chung-Ang University, Anseong 17546, Korea; 2 Korea Food Research Institute, Wanju 55365, Korea

**Keywords:** strawberry, volatile, gas chromatography-mass spectrometry, cold storage, maturity, modified atmosphere

## Abstract

‘Seolhyang’ strawberry is harvested before it is fully ripened and treated with CO_2_ to extend the shelf-life. However, the volatile changes in the ‘Seolhyang’ strawberry after short-term CO_2_ treatment have not been investigated, although the volatile profile is an important quality attribute. Herein, we investigated the effect of short-term high CO_2_ treatment on the changes in the composition of volatile compounds in ‘Seolhyang’ strawberries at two ripening stages (i.e., half-red and bright-red) during cold storage using headspace solid-phase microextraction and gas chromatography-mass spectrometry. Furthermore, the effect of CO_2_ treatment on fruit quality with respect to the aroma was investigated. A total of 30 volatile compounds were identified. Storage increased the volatile compound concentrations, and the total concentration of volatiles in the CO_2_-treated strawberries was lower than that of the untreated strawberries during storage. The production of some characteristic strawberry volatiles (e.g., 4-methoxy-2,5-dimethyl-3(2*H*)-furanone) was inhibited in CO_2_-treated strawberries. However, CO_2_ treatment helped maintain the concentrations of hexanal and 2-hexenal, which are responsible for the fresh odor in strawberries. Interestingly, CO_2_ treatment suppressed the production of off-odor volatiles, acetaldehyde, and hexanoic acid during strawberry storage. Thus, short-term CO_2_ treatment may help maintain the fresh aroma of strawberries during cold storage.

## 1. Introduction

‘Seolhyang’ (*Fragaria × ananassa* Duch) strawberry was developed in 2005 by cross-breeding the ‘Akihime’ and ‘Red Pearl’ cultivars in Korea [1]. This cultivar, which has a sweet taste and a characteristic sweet aroma, accounted for 87.5% of the Korean strawberry production in 2017 [2]. The strawberry cultivar is exported from Korea to various Asian countries. In 2020, approximately 1602 and 1208 tons of strawberries were exported from Korea to Hong Kong and Singapore, respectively [3]. However, the ‘Seolhyang’ cultivar, owing to its soft texture, has a short shelf-life compared with other traditional strawberry cultivars, such as ‘Maehyang’ and ‘Keumhyang’ [4]. Thus, harvesting unripened strawberries and postharvest treatments may prolong the shelf-life of ‘Seolhyang’ strawberry to enable shipping over long distances [4].

The characteristic aroma of the strawberry influences consumer acceptance [5]. Volatile compounds are responsible for strawberry aroma, and the volatile composition of strawberries depends on the cultivar and agricultural practice (e.g., harvesting location) [6]. ‘Carezza’ strawberry cultivar contains higher concentrations of hexyl acetate (8.11 µg/kg) than those in the ‘Darselect’ and ‘Marmolada’ cultivars (3.48–4.10 µg/kg), which have higher methyl hexanoate and methyl butanoate concentrations than those in the ‘Carezza’ cultivar, despite being grown in the same region in Switzerland [7]. Furthermore, the γ-decalactone content of 10 strawberry cultivars (i.e., Rubygem, Fortuna, Festival, Calinda, FL 127, Plared, Sahara, Sabrina, Victory, and E-22) ranged from n.d. to 62.76 µg/kg depending on the cultivar [8]. Among the volatile compounds found in strawberries, 2,5-dimethyl-4-hydroxy-3(2*H*)-furanone, methyl butanoate, ethyl butanoate, ethyl hexanoate, and methyl butanoate are responsible for the characteristic volatile compounds of ‘Elsanta’ strawberry grown in Belgium [9]. 

Furthermore, the ripening stage also affects the volatile compound composition of strawberries, and the total volatiles usually increase after ripening [7]. Especially, butyl acetate, methyl 2-methyl butanoate, ethyl butanoate, propyl butanoate, isopropyl hexanoate, and 3-phenyl-1-propanol are produced during ripening [7]. Additionally, C6 aldehydes are formed when lipoxygenase and hydroperoxide lyase activity increase during strawberry ripening [10]. Moreover, the activity of other enzymes, such as alcohol acyltransferase in some cultivars, increases during ripening, affecting the volatile composition of strawberries [11]. When the fruits are wounded, the lipoxygenase and hydroperoxide lyase activity increase up to 1.2 and 2.0 fold, respectively, resulting in an increase in 3-hexenal and trans-2-hexanal production [12]. 

Additionally, the volatile composition of strawberries changes during storage. For example, β-citronellol, hexanoic acid, and ethyl butanoate concentrations increased 5.3, 11.6, and 5.6 folds, respectively, after 7 d of cold storage at 10 °C [13]. Postharvest treatment also affects the volatile composition of strawberries. A previous study reported that while some volatiles, such as octyl acetate, butyl benzene, and 1-methyl-2-propyl benzene were not detected in high O_2_ (90% O_2_/10% N_2_)-exposed strawberries, other volatiles, such as α-terpineol, were newly formed [14]. In addition, hexanal treatment during storage increased the alcohol percentage of strawberry volatiles at the end of their shelf-life [15]. Storage under high CO_2_ exposure is an effective postharvest treatment that maintains fruit quality [16]. Elevated CO_2_ was more effective in inhibiting strawberry aroma changes than elevated O_2_ [17]. ‘Diamante’ strawberries, stored under elevated CO_2_ (20 kpa), maintained their characteristic volatile profile for 11 d at 5 °C [16]. However, long-term CO_2_ treatment may produce an unpleasant aroma, color change, and a decrease in the sugar and organic acid concentrations of strawberries [18]. Thus, short-term CO_2_ treatment may be required to extend the shelf-life of strawberries without quality loss. In our previous study, short-term CO_2_-treated ‘Seolhyang’ strawberries at two ripening stages showed similar texture, color, and metabolite profiles with lower decay rates after cold storage compared with the control fruit [19]. However, the volatile changes in the ‘Seolhyang’ strawberry after short-term CO_2_ treatment have not been investigated, although the volatile profile is an important quality attribute of strawberries. To the best of our knowledge, this is the first study investigating changes in the volatile composition of ‘Seolhyang’ strawberries depending on the postharvest treatment (i.e., short-term high CO_2_ treatment), maturity, and storage conditions. 

Short-term CO_2_ treatment may help maintaining the fresh aroma of strawberries during cold storage. In this study, the effect of short-term high CO_2_ treatment on the volatile composition of ‘Seolhyang’ strawberries was investigated at two ripening stages (half-red: 80% ripeness; bright-red: 100% ripeness) during 9 d of cold storage. These two ripening stages were chosen because unripened strawberries are harvested to prolong the shelf-life to be shipped over long distance, and ripened strawberries are also harvested to be shipped to short distance. In addition, ‘Seolhyang’ strawberries were chosen because the cultivar accounted for 87.5% of Korean strawberry production [2]. 

## 2. Results and Discussion

### 2.1. Identification of Volatile Compounds in Strawberries

**Figure 1:**
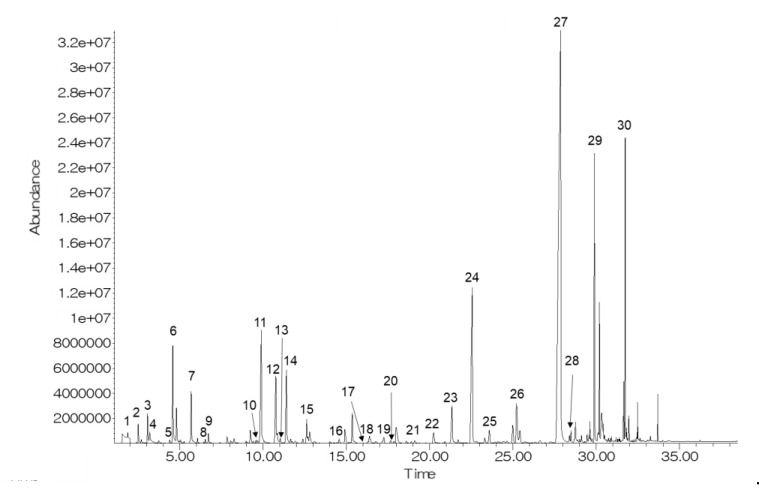
A representative GC chromatogram of CO_2_-treated bright-red strawberries after 9 d of storage at 10 °C. Volatile compound codes are provided in Table 1.

Figure 1 shows a representative GC chromatogram of CO_2_-treated bright-red strawberries after 9 d of storage. Thirty volatiles (5 aldehydes, 17 esters, 3 ketones, 2 alcohols, 2 acids, and 1 furanone) were identified (Table 1). Among the identified compounds, 11 volatile compounds (hexanal, 2-hexenal, nonanal, benzaldehyde, methyl acetate, methyl hexanoate, ethyl hexanoate, ethyl octanoate, 2-heptanone, butyric acid, and hexanoic acid) were identified by comparing them with the retention time and mass spectra of authentic standards. In previous studies, methyl butanoate, ethyl butanoate, methyl hexanoate, ethyl hexanoate, and 4-methoxy-2,5-dimethyl-3-(2H)-furanone were reported as characteristic volatiles of strawberries [20,21].

At day 0, some volatiles occurred or were present in higher concentrations only in the bright-red strawberries. For example, *(2E)*-hexenyl acetate was not present in half-red strawberries but was present in bright-red strawberries at day 0. *(2E)*-Hexenyl acetate was also reported in seven *Fragaria* × *ananassa* cultivars by Dong et al. [20]; however, it was not present in the ‘Camarosa’ and ‘Chandler’ strawberries harvested in India [22]. Thus, the volatile composition of strawberries may be affected by the cultivar type, maturity, and/or agricultural practices, such as the growing region. [23]. 

During cold storage, *(2E)*-hexenyl acetate and ethyl octanoate were newly produced in half-red and bright-red strawberries, respectively. The volatile composition of strawberries during cold storage was affected by the ripening stage when harvested. For example, ethyl octanoate was not found in fresh strawberries (day 0) during the two ripening stages; however, it was produced only in bright-red strawberries after cold storage, regardless of CO_2_ treatment. Previous studies found that ethyl octanoate was not present in strawberries of the ‘Cigaline’, ‘Chandler’, ‘Albion’, and ‘Festival’ cultivars [24,25,26]. Wang et al. reported that ethyl octanoate is formed after 5 d of storage in ‘Hong Yan’ strawberries [27]. The concentrations of some volatiles decreased or were absent after CO_2_ treatment. For example, the acetaldehyde content decreased, and 2-butanone was absent in bright-red strawberries after CO_2_ treatment.

### 2.2. Changes in the Composition of Volatile Compounds in Strawberries

**Figure 2:**
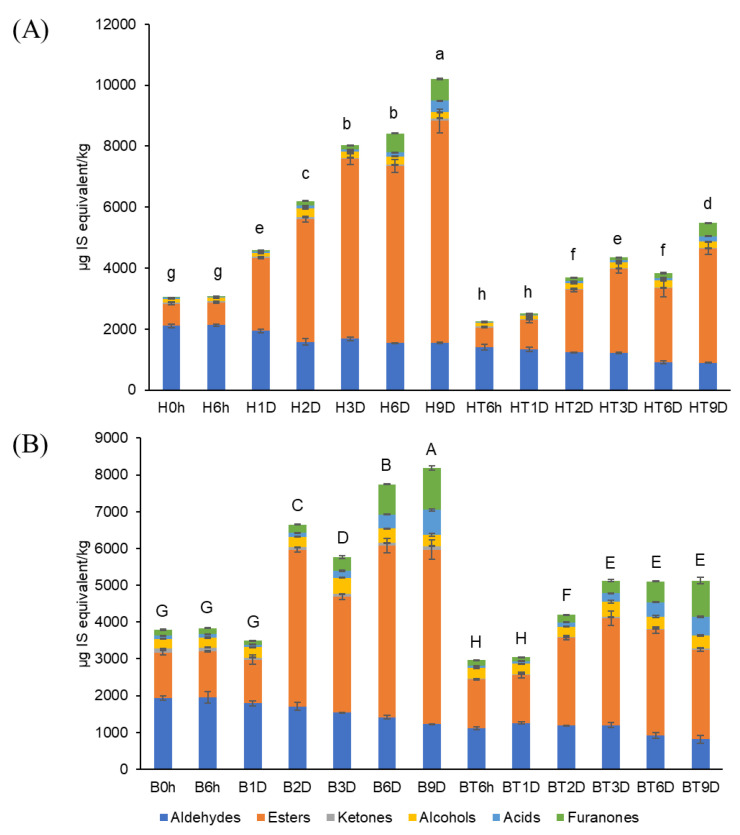
Sum of volatile compounds in (**A**) half-red and (**B**) bright-red strawberries stored at 10 °C for 9 d (H: half-red, B: bright-red, T: CO_2_-treated, h: hours, D: days). Lowercase and capital letters indicate significant difference for half-red and bright-red strawberries, respectively (*p* < 0.05).

Figure 2 shows the sum of the volatile compounds of the strawberries. Before storage (0 d), the sum of the volatiles in bright-red strawberries (B0h, 3787 µg/kg) was 1.24-fold higher than that of the half-red strawberries (H0h, 3044 µg/kg). These findings agree with those of previous studies, showing that the sum of the volatiles in ‘Cigaline’ strawberries increased during maturity [25]. After 9 d of storage, the volatile concentrations of strawberries used as control increased to a greater extent (B9D, 8,186 µg/kg; H9D, 10,198 µg/kg) than those of CO_2_-treated strawberries (BT9D, 5130 µg/kg; HT9D, 5480 µg/kg). Thus, short-term CO_2_ treatment of strawberries at two ripening stages may inhibit the production of volatiles during 9 d of storage. The MANOVA results showed that CO_2_ treatment and storage time affected the concentrations of the total volatiles (i.e., sum of volatiles) (*p* < 0.001), as shown in Supplementary Materials, Table S1.

The volatile composition of strawberries varied with maturity. For example, bright-red strawberries showed a higher ester/aldehyde ratio compared to that of half-red strawberries at day 0. After 9 d of storage, the aldehyde contents of half-red strawberries (H9D) and bright-red strawberries (B9D) decreased to 78% and 71%, respectively, compared to that at day 0. However, the ester contents of H9D and B9D increased 3.0-fold and 1.8-fold, respectively, compared to that at day 0. The ester content of CO_2_-treated half-red strawberries (HT9D) was 68.5% that of the total volatiles. This value was similar to that of the half-red.

**Figure 3:**
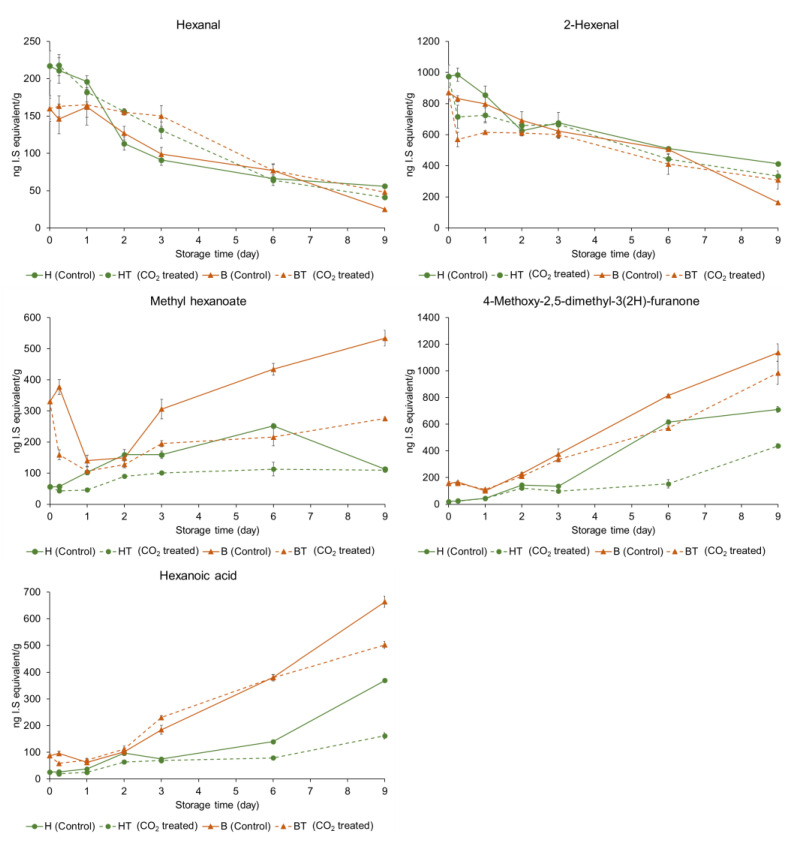
Comparisons of relative concentrations of characteristic volatile compounds in control and CO_2_-treated strawberries at varying stages of ripening stored at 10 °C for 9 d (H: half-red, B: bright-red, T: CO_2_ treated, h: hours, D: days). The concentration of each volatile compound was described as the relative concentration of the appropriate internal standard.

The relative concentrations of individual volatile compounds in the control and CO_2_-treated strawberries at two ripening stages—(A) half-red and (B) bright-red—during storage are shown in Table 2 and Table 3. The relative concentrations of characteristic volatile compounds of the strawberries are shown in Figure 3. The concentration of each volatile compound was described as the relative concentration of the appropriate internal standard. In half-red strawberries, the hexanal concentration significantly decreased from 217 µg/kg (H0h) to 41 µg/kg (HT9D) and 56 µg/kg (H9D) after 9 d in storage (*p* < 0.05). Furthermore, the 2-hexenal concentrations significantly decreased from 975 µg/kg (H0h) to 413 µg/kg (H9D) and 333 µg/kg (HT9D). No significant difference was observed in the hexanal and 2-hexenal concentrations between H9D and HT9D (*p* > 0.05). The hexanal concentrations of stored bright-red strawberries (B9D, 25 µg/kg; BT9D, 48 µg/kg) decreased compared to those in fresh bright-red strawberries (B0h, 160 µg/kg). Furthermore, the 2-hexenal concentrations of stored bright-red strawberries (B9D, 164 µg/kg; BT9D, 308 µg/kg) decreased compared to those of fresh half-red strawberries (B0h, 871 µg/kg). The 2-hexenal concentration of BT9D was significantly higher than that of B9D (*p* < 0.05). A previous study reported that the concentrations of hexanal and 2-hexenal, the characteristic volatile compounds responsible for the fresh and fruity odor of fresh strawberries, decreased during storage [28]. Hexanal may help delay the ripening process through the inactivation of phospholipase D, membrane lipid-degrading enzymes, and downstream oxidative signaling, and enhance shelf-life, as shown in mango fruits [29]. Moreover, hexanal may help reduce postharvest deterioration by preventing the growth of microorganism and fungi [30]. Interestingly, postharvest hexanal treatment on ‘Rubygem’ strawberries increased the alcohol percentage of the fruit at the end of their shelf-life [15]. However, in this study, short-term CO_2_ treatment on half-red strawberries did not increase the alcohol content at the end of the storage period. In this study, short-term CO_2_ treatment did not affect the hexanal and 2-hexenal concentrations in half-red strawberries but maintained their concentrations in bright-red strawberries. Thus, CO_2_ treatment on bright-red strawberries may help maintain fruit quality during storage. 

Characteristic strawberry volatile compounds include esters, such as ethyl butanoate, ethyl hexanoate, methyl butanoate, and methyl hexanoate [5]. Among them, methyl hexanoate showed a higher concentration than that of other esters. It has been previously suggested as a maturity marker for other strawberry cultivars (e.g., ‘Albion’, ‘Malling Pearl’, ‘Florin’, ‘Charlotte’, ‘Anabelle’, ‘Florin 2’, ‘Montery’, ‘San Andreas’, and ‘Portola’) [31]. The methyl hexanoate concentration of B0h (330 µg/kg) was 5.9-folds higher than that of H0h (56 µg/kg) before storage (day 0). The methyl hexanoate concentration of control half-red control strawberries increased and then decreased during storage. Ayala-Zavala et al. also showed that the methyl hexanoate concentration of commercially matured ‘Chandler’ strawberries increased 1.8-fold when stored at 5 °C for 5 d, but it decreased to the concentrations lower than the original concentration when harvested [32]. However, the methyl hexanoate concentration continued to increase at a slower rate in CO_2_-treated half-red (HT) strawberries but not in the in control half-red strawberries. The methyl hexanoate concentrations decreased during early storage (≈2 d) and then increased both in the control and CO_2_-treated bright-red strawberries. The concentrations decreased to a greater extent in CO_2_-treated bright-red strawberries until 2 d of storage and increased at a slower rate in CO_2_-treated bright-red strawberries compared to the control bright-red strawberries stored under the same conditions. Thus, CO_2_ treatment may induce a decline in the methyl hexanoate concentration during the early storage period and then inhibit methyl hexanoate formation during storage, regardless of the ripening stage. Interestingly, the production of methyl butanoate, ethyl butanoate, and ethyl hexanoate was inhibited in CO_2_-treated half-red strawberries during storage; however, the effect was not observed or was less frequently observed in bright-red strawberries. The concentration of esters in CO_2_-treated bright-red strawberries was 81–98% that of the control at 9 d of storage. 

The 4-methoxy-2,5-dimethyl-3(2H)-furanone concentration of bright-red strawberries (B0h, 156 µg/kg) was 7.8-fold higher than that of half-red strawberries (H0h, 20 µg/kg). Furanones, such as 2,5-dimethyl-4-hydroxy-3(2H)-furanone and 4-methoxy-2,5-dimethyl-3(2H)-furanone, are known as characteristic volatile compounds of strawberry [5]. However, 2,5-dimethyl-4-hydroxy-3(2H)-furanone was not detected in the ‘Seolhyang’ strawberry. The concentration of 4-methoxy-2,5-dimethyl-3(2H)-furanone of half-red strawberries (H0h, 20 µg/kg) increased 35.6-folds after 9 d of storage (H9D, 711 µg/kg). The concentration of 4-methoxy-2,5-dimethyl-3(2H)-furanone of bright-red strawberries (B0h, 156 µg/kg) increased by 7.3-folds after 9 d storage (B9D, 1,137 µg/kg). For the CO_2_ treatment groups, the 4-methoxy-2,5-dimethyl-3(2H)-furanone concentrations also increased but were lower compared to the control groups (H9D (711 µg/kg) vs. HT9D (411 µg/kg); B9D (1,137 µg/kg) vs. BT9D (985 µg/kg)). Thus, CO_2_ treatment may inhibit the production of 4-methoxy-2,5-dimethyl-3(2H)-furanone during storage. 

High CO_2_ treatment of strawberries may induce a decrease in alcohol acetyltransferase activity, which is responsible for ester biosynthesis from acetyl CoA [33]. Thus, short-term CO_2_ treatment of half-red and bright-red strawberries may inhibit the production of esters during storage. In a previous study, CO_2_ treatment of strawberries inhibited the production of glucose, which is a carbon source for 4-hydroxy-2,5-dimethyl-3(2H)-furanone [34,35]. The concentrations of furanones, such as 4-methoxy-2,5-dimethyl-3(2H)-furanone, increased during postharvest ripening possibly because of an increase in *Fragaria* × *ananassa* O-methyltransferase (FaOMT) activity that changes methylated 4-hydroxy-2,5-dimethyl-3(2H)-furanone into 4-methoxy-2,5-dimethyl-3(2H)-furanone [36,37]. Thus, CO_2_ treatment may also inhibit furanone accumulation during storage by decreasing the glucose concentration, which is responsible for furanone biosynthesis and decreasing FaOMT activity during storage. 

Acetaldehyde is responsible for the pungent aroma, which is the off-odor in strawberries [38]. The acetaldehyde concentration of half-red strawberries (H0h, 899 µg/kg) was similar to that of bright-red strawberries (B0h, 881 µg/kg). The acetaldehyde concentration of half-red strawberries (H0h, 899 µg/kg) increased after 9 d of storage (H9D, 986 µg/kg). In the case of bright-red strawberries, the acetaldehyde concentration (B0h, 881 µg/kg) increased after 9 d of storage (B9D, 988 µg/kg). The acetaldehyde concentration of H9D (986 µg/kg) was 2.0-fold higher than that of HT9D (505 µg/kg). The acetaldehyde concentration of B9D (988 µg/kg) was 2.2-fold higher than that of BT9D (440 µg/kg). Thus, CO_2_ treatment may inhibit acetaldehyde formation and help maintain the aroma quality of strawberries during storage, regardless of maturity. A previous study reported that the acetaldehyde content was lower in strawberries (*Fragaria vesca* L.) stored in a controlled atmosphere with a higher CO_2_ concentration compared to that in the untreated control [38]. High CO_2_ treatment inhibited pyruvate decarboxylase activity that decarboxylates pyruvate to yield acetaldehyde during storage [39]. 

2,3-Butanedione is responsible for the pungent off-odor of strawberries [40]. The 2,3-butanedione concentration of bright-red strawberries (B0h, 92 µg/kg) was 2.9-fold higher than that of half-red strawberries (H0h, 32 µg/kg). The 2,3-butanedione concentration was lower in CO_2_-treated strawberries during storage compared to untreated strawberries at the same storage time, regardless of the ripening stage. Thus, CO_2_ treatment may improve the aroma quality of strawberries during storage regardless of the maturity based on lower concentrations of 2,3-butanedione compared to the control during storage.

Hexanoic acid is also related to a rancid off-odor in strawberries [40]. The concentration of hexanoic acid of half-red strawberries increased from 26 µg/kg (H0h) to 370 µg/kg (H9D) and 162 µg/kg (HT9D). In bright-red strawberries, the concentration of hexanoic acid increased from 87 µg/kg (B0h) to 664 µg/kg (B9D) and 502 µg/kg (BT9D). Thus, hexanoic acid concentrations increased during storage regardless of the ripening stage; however, CO_2_ treatment may inhibit hexanoic acid production during storage. Similar to our findings, the hexanoic acid concentrations of (untreated) ‘Sweet Charlie’ strawberries increased during 9 d storage at 4 °C [10]. However, the hexanoic acid concentrations of ‘Pajaro’ strawberries decreased during 12 d storage in 20% CO_2_ at 0 °C [41]. In addition to hexanoic acid, various fatty acids, including linoleic acid and linolenic acid, were identified in nine strawberry cultivars in a previous study [42]. Some ester compounds, such as methyl butanoate, ethyl butanoate, and methyl hexanoate, are formed by the β-oxidation of fatty acids in strawberries [18]. In this study, the ester compound production may have been inhibited during storage by CO_2_ treatment possibly owing to inhibited fatty acid oxidation during storage.

### 2.3. Principal Component Analysis (PCA) and the Heatmap of Volatiles

**Figure 4:**
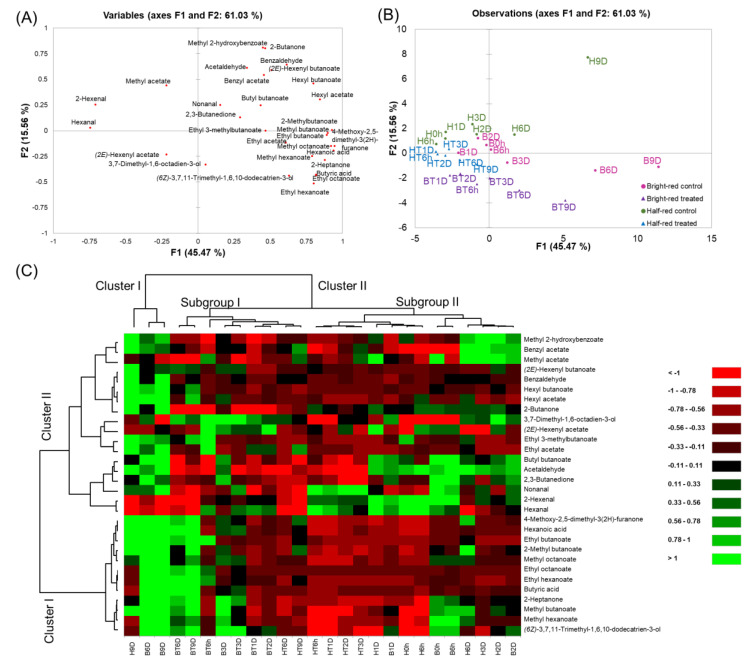
(**A**) Score plot, (**B**) loading plot of the principal components analysis, and (**C**) hierarchical clustering and heatmap visualization of the changes in the volatile compounds in strawberries during storage. (H: half-red, B: bright-red, T: CO_2_ treated, h: hours, D: days). Green and red colors presented in the heatmap indicate values above and below the mean centered and scaled expression values, respectively. Black indicates values close to the mean. The total number of features was the same as the number of determined volatiles in the strawberries (n = 30), and the “non-specific filtering” option in the XLSTAT program was not used. Missing data were estimated using the nearest-neighbor algorithm. Hierarchical clustering was conducted based on Euclidian distances.

A PCA was performed using the volatile compounds data from all samples (Figure 4A and B). The first two principal components (PC1 and PC2) accounted for 61.03% of the total variation. PC1 and PC2 contributed to 45.47% and 15.56% of the total variation, respectively. The control strawberries in mostly late storage periods (H9D, B6D, and B9D) were separately grouped from other samples. As storage progressed, the strawberries were spread on the positive side of PC1. They were positively correlated with characteristic strawberry volatiles, such as 4-methoxy-2,5-dimethyl-3(2*H*)-furanone, methyl butanoate, ethyl butanoate, methyl hexanoate, and ethyl hexanoate. Acetaldehyde and 2,3-butanedione, and hexanoic acid, that are responsible for the off-odor of strawberries, were spread on the positive side of PC1. These volatiles correlated with H9D, B6D, and B9D. Hexanal and 2-hexenal, that are responsible for the fruity odor of strawberries, were spread on the negative side of PC1. These volatiles correlated with the half-red strawberries during the early storage period (e.g., H0h, H6h, H1D, HT6h, and HT1D). Additionally, CO_2_-treated strawberries were spread on the negative side of PC2. Especially, CO_2_-treated bright-red strawberries were spread toward the negative side of PC2 compared to CO_2_-treated half-red strawberries. *(2E)*-2-hexenyl acetate and 3,7-dimethyl-1,6-octadien-3-ol concentrations positively correlated with CO_2_-treated strawberry groups. Thus, these may be used as potential markers of short-term CO_2_ treatment in strawberries. Previously, 3,7-dimethyl-1,6-octadien-3-ol was reported as a key component of the muscat table grape [43]. The concentration of 3,7-dimethyl-1,6-octadien-3-ol was higher in CO_2_-treated strawberry groups compared to the control groups, regardless of the ripening stage. Ten volatiles (4-methoxy-2,5-dimethyl-3(2*H*)-furanone, hexanoic acid, methyl octanoate, methyl butanoate, 2-heptanone, ethyl butanoate, butyric acid, hexyl acetate, ethyl octanoate, and hexyl butanoate) were selected from the loading plot based on their high variables importance in projection (VIP) values over 1, as they contributed to the discrimination of the strawberries samples.

A dendrogram was obtained using cluster analysis (Figure 4C). Volatiles were grouped into two clusters. Cluster I included the characteristic strawberry volatiles (e.g., 4-methoxy-2,5-dimethyl-3(2*H*)-furanone, ethyl butanoate, ethyl hexanoate, methyl butanoate, and methyl hexanoate) showing higher concentrations in bright-red strawberries, regardless of CO_2_ treatment. Strawberries were grouped into two clusters. Cluster I included control strawberries at the late storage period (i.e., H9D, B6D, and B9D). Cluster II was divided into two subgroups. Subgroup I included CO_2_-treated half-red and bright-red strawberries, except B3D. Subgroup II included control half-red and bright-red strawberries, except CO_2_-treated half-red strawberries in the early storage period (i.e., HT6h-HT3D).

## 3. Materials and Methods

### 3.1. Chemicals and Reagents 

Authentic standards of hexanal, 2-hexenal, nonanal, benzaldehyde, methyl acetate, methyl hexanoate, ethyl hexanoate, ethyl octanoate, 2-heptanone, butyric acid, hexanoic acid, and n-alkane standards were purchased from Sigma-Aldrich (St. Louis, MO, USA). Authentic standards of octanal-d16, ethyl butanoate-d3 and n-hexyl-d13 alcohol, used as internal standards, were purchased from C/D/N Isotope (Pointe-Claire, Quebec, Canada). Nanopure water used in the study was obtained from a water purification system (Milli-Q Direct 8, Merck Millipore, Billerica, MA, USA).

### 3.2. Strawberry Samples 

‘Seolhyang’ strawberries were harvested at two different ripening stages from a strawberry farm located in Jinju, Gyeongsangnam-do, Korea (35.1800 °N, 128.1076 °E) in March 2019. The ‘Seolhyang’ strawberries were grown in a commercial greenhouse using conventional soil cultivation practices. The samples were transported to the Korea Food Research Institute (KFRI; Wanju, Korea), which was located at a distance of 175 km from the farm, using a refrigerated truck. Undamaged strawberries of uniform size and shape were selected for the study. The ripening stages were grouped into half-red (80% maturity) or bright-red (100% maturity) based on surface color development. Half of the selected fruits under each ripening group were treated with CO_2_ by exposing the samples to air saturated with 30% CO_2_ and 70% N_2_ for 3 h at 25 °C. Thus, the samples were divided into four subgroups (i.e., half-red control, half-red CO_2_-treated, bright-red control, and bright-red CO_2_-treated). Approximately 15–20 fruits from each subgroup were placed in a plastic bin and covered with a lid. The two bins were removed periodically at 0, 6, 24, and 48 h, and 3, 6, and 9 d to sample the strawberries stored at 10 °C. To compare the changes in the volatile profiles of strawberries following CO_2_ treatment, the volatile composition of strawberries after 6 h was investigated. Thus, there were 8 (time point) × 2 (half-red and bright-red) × 2 (control and CO_2_ treated) × 2 (duplicate) = 64 samples for strawberries stored at 10 °C. Each of the samples at the time points were immediately frozen in liquid nitrogen and stored at −80 °C until analysis.

### 3.3. Determination of Strawberry Volatiles using HS-SPME-GC/MS 

The sample preparation method was adopted from our previous study with modifications [44]. Frozen strawberries (300 g) were ground using a blender (BL682 Auto-IQ, Nutri Ninja, Boston, MA, USA) for 1 min. Then, 5 g of the ground frozen strawberries was transferred to a glass headspace vial (Gerstel, Baltimore, MD, USA), and 5 mL of saturated sodium chloride solution was added. An internal standard mixture (octanal-d16, ethyl butanoate-d3, and n-hexyl-d13 alcohol) was added to the glass headspace vial with a final concentration of 10 µg/kg and was sealed immediately using a magnetic crimp cap (Gerstel).

After 10 min of equilibration, a 2 cm solid phase microextraction (SPME) fiber coated with divinylbenzene/carboxen/ polydimethylsiloxane (DVB/CAR/PDMS) was used to extract the volatiles. The volatiles were absorbed for 30 min at 40 °C and desorbed for 30 s at 250 °C. Volatiles in strawberry were determined using gas chromatography (7890B, Agilent technologies, Santa Clara, CA, USA) coupled with a mass selective detector (5977B, Agilent technologies) and separated on a DB-WAX capillary column (30 m × 0.25 mm i.d., 0.25 µm film thickness; Agilent technologies). The oven temperature was set to 40 °C for 2 min; the temperature was increased by 4 °C/min to 120 °C and held at 120 °C for 5 min. Then, the temperature was increased to 250 °C at a rate of 20 °C/min and held at 250 °C for 5 min. The solvent delay was set to 3 min. Helium (99.999%) was used as a carrier gas at a flow rate of 1 mL/min. The inlet temperature was 250 °C, and an inlet liner (0.75 mm i.d., Agilent technologies) was used for the splitless mode. The temperatures of the transfer line, ion source, and quadrupole were 240 °C, 230 °C, and 150 °C, respectively. The electron energy was 70 eV. Mass spectra were obtained in the range of *m/z* 30–350 at a rate of 3.06 scans/s.

### 3.4. Identification and Relative Quantification of Volatiles in Strawberries

Volatiles were identified by comparing their mass spectra and the retention time of authentic standards if available or tentatively by searching libraries (Willey and NIST 08 mass spectral libraries) for compounds with no authentic standard available. Eighty percent of the match score was used as a cut-off, and Kovats’ retention index was used to confirm the identification of volatiles. Kovats’ retention index was calculated using the retention time of each volatile compound and an authentic alkane standard mixture.

Relative volatile concentrations were calculated according to formula (1) presented below [45]. Briefly, the extracted ion peak area of each volatile was divided by the extracted ion peak area of the respective internal standard (octanal-d16 for aldehydes, ketones, and furanones; n-hexyl-d13 alcohol for alcohols and acids; ethyl butanoate-d3 for esters). The relative concentration of each volatile was converted by multiplying the area ratio with 10 µg internal standard/kg strawberry. The results were expressed on a fresh weight basis.

(1) Concentration µgkg=extracted ion peak areaextracted ion peak area of internal standardinternal standard 10 µgkg

### 3.5. Statistical Analyses 

Statistical analyses were conducted using SPSS statistics 23 software (SPSS, Inc., Chicago, IL, USA). Significant differences in the volatile composition among the strawberry fruits were confirmed by an analysis of variance at *p* < 0.05. Duncan’s test was used as a post hoc analysis. A multivariate analysis of variance (MANOVA) was conducted to evaluate the effects of maturity, storage time, CO_2_ treatment, and their interactions on the volatile composition of strawberries. PCA, heatmap, and hierarchical cluster analyses were performed using XLSTAT (XLSTAT, ver. 2017.03, Microsoft Excel Add-in software, New York, NY, USA). The data format for PCA was an observation/variable table, and Pearson’s correlation type was used as the PCA type. Based on the volatile profile of strawberries, heatmap and hierarchical cluster analyses were conducted to demonstrate similarities and differences among the strawberries depending on maturity, storage period, and short-term CO_2_ treatment. The total number of features was the same as the number of determined volatiles in the strawberries (n = 30), and the “non-specific filtering” option in the XLSTAT program was not used. 

## 4. Conclusions

To the best of our knowledge, this is the first study investigating the effect of short-term CO_2_ treatment on the volatile composition of ‘Seolhyang’ strawberries. A total of thirty volatiles were identified using HS-SPME with GC-MS in ‘Seolhyang’ strawberries stored at 10 °C for 9 d. The volatile composition of the strawberries changed depending on the ripening stages, storage time, and short-term CO_2_ treatment. Ripening and storage increased the total volatile concentrations and CO_2_ treatment inhibited the production of volatiles. The production of some characteristic strawberry volatiles, such as methyl hexanoate and 4-methoxy-2,5-dimethyl-3(2*H*)-furanone, was inhibited in CO_2_-treated strawberries, especially in half-red strawberries, during cold storage. However, CO_2_ treatment helped maintain the hexanal and 2-hexenal concentrations, which are responsible for the fresh odor in strawberries. Interestingly, CO_2_ treatment decreased the formation of rancid off-odor volatiles, such as acetaldehyde, 2,3-butanedione, and hexanoic acid during storage, regardless of the ripening state. Thus, short-term CO_2_ treatment may help maintain a fresh odor and reduce the off-odor, improving strawberry aroma characteristics during storage. Short-term CO_2_ treatment may be applied to ‘Seolhyang’ strawberries for long-distance shipping to improve the strawberry aroma characteristics during shipping. In future research, the role of short-term CO_2_ treatment in improving the aroma quality of ‘Seolhyang’ strawberries can be confirmed using descriptive sensory analysis.

## Figures and Tables

**Table 1:** Volatile compounds identified using HS-SPME-GC/MS in strawberries.

Peak no.	Volatile Compound	Odor Description ^a^	DB-WAX Column		DB-5MS Column		Extracted Ion (*m/z*)^c^	Internal Standard	Compound Newly Produced During Storage
			Unknown RI	Literature RI^b^	Unknown RI	Literature RI^b^			
*Aldehydes*									
1	Acetaldehyde	Pungent	660	677			32	Octanal-*d_16_*	
9	Hexanal ^*^	Fresh, green	1073	1077	801	802	56	Octanal- *d_16_*	
12	2-Hexenal ^*^	Fruity, green	1204	1205	856	854	41	Octanal- *d_16_*	
18	Nonanal ^*^	Citrus	1378	1375			57	Octanal- *d_16_*	
22	Benzaldehyde ^*^	Fruity, nutty	1496	1495	967	961	106	Octanal- *d_16_*	
*Esters*									
2	Methyl acetate ^*^	Fruity	811	813			42	Ethyl butanoate-*d_3_*	
3	Ethyl acetate	Fruity, sweet	866	867			43	Ethyl butanoate-*d_3_*	
6	Methyl butanoate	Fruity, apple	976	971	653	697	74	Ethyl butanoate-*d_3_*	
7	Ethyl butanoate	Apple	1032	1037			71	Ethyl butanoate-*d_3_*	
8	Ethyl 3-methylbutanoate	Sweet, fruity	1062	1064	841	856	88	Ethyl butanoate-*d_3_*	
11	Methyl hexanoate^*^	Fruity, pineapple	1181	1183	925	910	74	Ethyl butanoate-*d_3_*	
13	Butyl butanoate	Sweet, fruity, fresh	1207	1208			71	Ethyl butanoate-*d_3_*	
14	Ethyl hexanoate ^*^	Fruity, sweet, pineapple	1227	1220	997	999	88	Ethyl butanoate-*d_3_*	
15	Hexyl acetate	Green, fruity, apple	1263	1261	1011	1011	43	Ethyl butanoate-*d_3_*	
16	*(2E)*-Hexenyl acetate	Sweet, leafy, green	1321	1322	1014	1025	43	Ethyl butanoate-*d_3_*	New
17	Methyl octanoate	Green, sweet, orange	1377	1378	1122	1120	74	Ethyl butanoate-*d_3_*	
19	Hexyl butanoate	Green, sweet, fruity	1401	1407	1187	1191	43	Ethyl butanoate-*d_3_*	
20	Ethyl octanoate ^*^	Sweet, pineapple	1421	1420			88	Ethyl butanoate-*d_3_*	New
21	*(2E)*-Hexenyl butanoate	Ripe, fruity	1461	1461			71	Ethyl butanoate-*d_3_*	
26	2-Methylbutanoate	Acidic, fruity	1647	1641	849	854	74	Ethyl butanoate-*d_3_*	
27	Benzyl acetate	Sweet, fruit, floral	1708	1710	1161	1170	108	Ethyl butanoate-*d_3_*	
28	Methyl 2-hydroxybenzoate	Sweet, aromatic	1761	1762	1192	1193	120	Ethyl butanoate-*d_3_*	
*Ketones*									
4	2-Butanone	Ethereal	878	889			43	Octanal-*d_16_*	
5	2,3-Butanedione	Pungent	965	965			43	Octanal-*d_16_*	
10	2-Heptanone ^*^	Fruity, green	1172	1178	890	889	43	Octanal-*d_16_*	
*Alcohols*									
23	3,7-Dimethyl-1,6-octadien-3-ol	Citrus, orange, floral	1534	1534	1100	1101	71	Hexyl-*d_13_* alcohol	
30	*(6Z)*-3,7,11-Trimethyl-1,6,10-dodecatrien-3-ol	Mild floral	2033	2033	1559	1566	69	Hexyl-*d_13_* alcohol	
*Acids*									
25	Butyric acid ^*^	Sharp, dairy-like, cheesy	1604	1607			60	Hexyl-*d_13_* alcohol	
29	Hexanoic acid ^*^	Sour, rancid, sweat	1832	1834	985	990	60	Hexyl-*d_13_* alcohol	
*Furanones*									
24	4-Methoxy-2,5-dimethyl-3(2*H*)-furanone	Sweet, caramel	1571	1580	1059	1057	142	Octanal-*d_16_*	

^a^ Odor description of volatile compounds was obtained from https://www.flavornet.org. ^b^ RI indicates retention index. RI values were obtained from https://www.nist.gov. ^c^ Extracted ion from total ion scan used for quantification of each volatiles. * Indicates that identification of the volatile compound was confirmed by comparing their retention times and the mass spectra of their authentic standards.

**Table 2:** Changes in the relative concentrations of volatile compounds in untreated (control) and CO_2_-treated half-red strawberries.

Volatile Compound	H0h			H6h			H1D			H2D			H3D			H6D			H9D			HT6h			HT1D			HT2D			HT3D			HT6D			HT9D		
*Aldehydes*																																							
Acetaldehyde	899 ± 31 bc			923 ± 25 bc			874 ± 46 cd			821 ± 62 d			898 ± 14 bc			941 ± 2 ab			986 ± 14 a			464 ± 12 ef			413 ± 8 fg			410 ± 5 fg			403 ± 7 g			393 ± 10 g			505 ± 2 e		
Hexanal	217 ± 20 a			211 ± 17 a			196 ± 8 ab			113 ± 9 de			91 ± 7 e			66 ± 3 f			56 ± 2 fg			218 ± 14 a			182 ± 13 b			156 ± 4 c			131 ± 11 cd			64 ± 7 fg			41 ± 0 g		
2-Hexenal	975 ± 73 a			985 ± 43 a			854 ± 1 b			625 ± 33 d			676 ± 67 cd			511 ± 6 e			413 ± 6 fg			715 ± 74 cd			725 ± 49 c			659 ± 8 cd			666 ± 12 cd			444 ± 28 ef			333 ± 8 g		
Nonanal	4 ± 0 e			4 ± 0 e			5 ± 1 c			4 ± 0 de			6 ± 0 c			5 ± 1 cd			6 ± 0 c			7 ± 1 b			7 ± 0 b			7 ± 0 b			8 ± 0 a			4 ± 0 e			2 ± 0 f		
Benzaldehyde	12 ± 2 de			8 ± 1 fg			9 ± 1 f			12 ± 1 e			14 ± 0 cd			17 ± 0 b			88 ± 1 a			8 ± 1 fg			6 ± 0 g			12 ± 1 e			9 ± 1 f			14 ± 1 cde			15 ± 1 bc		
*Esters*																																							
Methyl acetate	12 ± 1 d			10 ± 1 e			21 ± 0 a			22 ± 2 a			18 ± 0 b			18 ± 0 b			13 ± 1 d			13 ± 2 d			11 ± 0 de			12 ± 0 d			15 ± 0 c			12 ± 0 d			15 ± 0 c		
Ethyl acetate	11 ± 2 e			17 ± 1 d			5 ± 0 h			6 ± 0 gh			5 ± 0 hi			30 ± 1 c			63 ± 3 a			8 ± 0 efg			7 ± 1 fgh			9 ± 0 ef			1 ± 0 j			2 ± 0 ij			48 ± 1 b		
Methyl butanoate	53 ± 1 g			50 ± 2 g			93 ± 6 e			131 ± 3 c			110 ± 7 d			177 ± 4 b			192 ± 3 a			54 ± 1 g			40 ± 1 h			58 ± 4 g			77 ± 7 f			70 ± 5 f			89 ± 4 e		
Ethyl butanoate	5 ± 0 cd			4 ± 0 cd			2 ± 0 d			5 ± 0 cd			4 ± 0 cd			42 ± 0 b			159 ± 11 a			10 ± 1 c			6 ± 0 cd			5 ± 0 cd			8 ± 0 cd			8 ± 1 cd			36 ± 1 b		
Ethyl 3-methylbutanoate	3 ± 1 b			n.d.			n.d.			n.d.			n.d.			2 ± 0 c			5 ± 0 a			1 ± 0 d			n.d.			n.d.			n.d.			n.d.			n.d.		
Methyl hexanoate	56 ± 4 e			57 ± 4 e			103 ± 1 cd			159 ± 17 b			159 ± 12 b			252 ± 8 a			113 ± 4 c			43 ± 3 e			46 ± 1 e			90 ± 5 d			101 ± 1 cd			113 ± 22 c			110 ± 2 cd		
Butyl butanoate	15 ± 3 abc			13 ± 0 cdef			18 ± 0 a			17 ± 3 ab			15 ± 1 bcd			16 ± 1 abc			14 ± 1 bcd			14 ± 1 bcde			11 ± 1 ef			10 ± 0 fg			11 ± 1 def			12 ± 1 def			7 ± 0 g		
Ethyl hexanoate	2 ± 0 ef			3 ± 0 d			1 ± 0 gh			2 ± 0 e			1 ± 0 fgh			7 ± 1 b			6 ± 0 c			1 ± 0 fgh			1 ± 0 h			1 ± 0 fgh			1 ± 0 gh			2 ± 0 efg			27 ± 1 a		
Hexyl acetate	21 ± 1 d			28 ± 3 c			13 ± 0 ef			9 ± 1 gh			5 ± 0 i			31 ± 0 b			119 ± 3 a			15 ± 1 e			6 ± 0 hi			7 ± 0 hi			4 ± 1 i			11 ± 2 fg			12 ± 1 efg		
*(2E)*-hexenyl acetate	n.d.			20 ± 0 b			31 ± 1 a			18 ± 1 c			5 ± 0 g			5 ± 0 g			8 ± 0 f			14 ± 2 d			9 ± 1 ef			17 ± 1 c			6 ± 0 g			8 ± 0 f			10 ± 1 e		
Methyl octanoate	2 ± 0 d			1 ± 0 e			1 ± 0 e			5 ± 1 b			4 ± 0 c			7 ± 0 a			8 ± 0 a			1 ± 0 e			n.d.			n.d.			3 ± 0 d			4 ± 0 c			4 ± 0 c		
Hexyl butanoate	1 ± 0 b			1 ± 0 b			1 ± 0 b			2 ± 0 b			1 ± 0 b			2 ± 0 b			18 ± 3 a			1 ± 0 b			1 ± 0 b			1 ± 0 b			1 ± 0 b			1 ± 0 b			n.d.		
*(2E)*-Hexenyl butanoate	1 ± 0 fgh			1 ± 0 gh			1 ± 0 ef			3 ± 0 b			1 ± 0 hi			1 ± 0 fg			16 ± 0 a			2 ± 0 c			2 ± 0 d			1 ± 0 de			1 ± 0 i			1 ± 0 de			1 ± 0 ef		
2-Methyl butanoate	22 ± 3 ef			24 ± 1 ef			27 ± 1 e			50 ± 5 d			44 ± 1 d			161 ± 9 a			123 ± 9 b			26 ± 0 ef			18 ± 1 ef			24 ± 1 ef			17 ± 1 f			26 ± 4 ef			86 ± 2 c		
Benzyl acetate	519 ± 43 g			506 ± 32 g			2067 ± 7 f			3608 ± 65 d			5519 ± 150 b			5050 ± 229 c			6414 ± 394 a			436 ± 11 g			804 ± 76 g			1801 ± 57 f			2521 ± 141 e			2161 ± 253 ef			3303 ± 194 d		
Methyl 2-hydroxybenzoate	3 ± 1 cde			1 ± 0 f			2 ± 0 de			4 ± 0 c			5 ± 0 b			3 ± 0 cd			7 ± 1 a			1 ± 0 ef			1 ± 0 f			2 ± 0 ef			2 ± 0 ef			2 ± 0 ef			2 ± 0 ef		
*Ketones*																																							
2-Butanone	12 ± 1 def			16 ± 2 bcd			18 ± 1 b			16 ± 2 bc			14 ± 0 bcde			15 ± 0 bcde			48 ± 4 a			14 ± 1 cde			13 ± 1 cdef			10 ± 0 fg			11 ± 1 ef			6 ± 0 h			6 ± 0 gh		
2,3-Butanedione	32 ± 2 c			32 ± 3 c			25 ± 1 d			39 ± 0 a			22 ± 1 d			40 ± 2 a			35 ± 0 b			24 ± 2 d			19 ± 1 e			13 ± 1 f			8 ± 0 g			14 ± 1 f			15 ± 1 f		
2-Heptanone	2 ± 0 d			1 ± 0 i			1 ± 0 h			6 ± 0 b			5 ± 0 c			6 ± 0 a			6 ± 0 a			2 ± 0 ef			2 ± 0 ef			2 ± 0 gh			2 ± 0 fg			2 ± 0 fg			2 ± 0 e		
*Alcohols*																																							
3,7-Dimethyl-1,6-octadien-3-ol	82 ± 7 f			85 ± 0 f			101 ± 2 e			157 ± 8 a			130 ± 0 bc			138 ± 3 b			111 ± 4 de			90 ± 8 f			81 ± 1 f			125 ± 4 c			123 ± 1 c			127 ± 11 bc			120 ± 1 cd		
*(6Z)*-3,7,11-Trimethyl-1,6,10-dodecatrien-3-ol	35 ± 3 ef			36 ± 1 ef			27 ± 0 f			123 ± 9 a			64 ± 4 d			118 ± 10 a			90 ± 8 bc			39 ± 2 ef			33 ± 2 f			76 ± 1 cd			50 ± 2 e			102 ± 16 b			88 ± 0 bc		
*Acids*																																							
Butyric acid	1 ± 0 c			n.d.			1 ± 0 ef			2 ± 0 a			1 ± 0 d			2 ± 0 b			n.d.			n.d.			n.d.			1 ± 0 g			1 ± 0 fg			1 ± 0 de			1 ± 0 ef		
Hexanoic acid	26 ± 3 h			27 ± 0 h			38 ± 0 g			97 ± 10 d			75 ± 4 ef			140 ± 5 c			370 ± 2 a			19 ± 1 h			25 ± 0 h			64 ± 3 f			69 ± 2 ef			79 ± 3 e			162 ± 12 b		
*Furanones*																																							
4-Methoxy-2,5-dimethyl-3(2H)-furanone	20 ± 1 g			26 ± 1 g			45 ± 1 g			145 ± 12 de			135 ± 10 de			617 ± 17 b			711 ± 22 a			24 ± 1 g			44 ± 0 g			121 ± 11 ef			98 ± 4 f			153 ± 31 d			437 ± 18 c		
Sum	3044 ± 99 g			3090 ± 53 g			4584 ± 70 e			6199 ± 235 c			8027 ± 132 b			8420 ± 184 b			10198 ± 380 a			2263 ± 104 h			2511 ± 14 h			3694 ± 80 f			4348 ± 162 e			3835 ± 372 f			5480 ± 181 d		

Different letters within the same line denote significant differences in the same compound (*p* < 0.05).

**Table 3:** Changes in the relative concentrations of volatile compounds in untreated (control) and CO_2_-treated bright-red strawberries.

Volatile Compound	B0h			B6h			B1D			B2D			B3D			B6D			B9D			BT6h			BT1D			BT2D			BT3D			BT6D			BT9D		
*Aldehydes*																																							
Acetaldehyde	881 ± 9 abc			954 ± 134 ab			824 ± 65 c			872 ± 35 bc			802 ± 49 c			811 ± 29 c			988 ± 2 a			369 ± 5 d			464 ± 9 d			402 ± 13 d			430 ± 49 d			420 ± 5 d			440 ± 36 d		
Hexanal	160 ± 14 ab			146 ± 30 ab			162 ± 24 ab			127 ± 9 bc			99 ± 9 cd			77 ± 9 de			25 ± 0 f			163 ± 17 ab			165 ± 14 a			155 ± 17 ab			150 ± 4 ab			77 ± 14 de			48 ± 8 ef		
2-Hexenal	871 ± 41 a			831 ± 20 a			798 ± 114 a			692 ± 55 b			623 ± 43 bc			506 ± 15 cd			164 ± 8 f			569 ± 47 c			615 ± 10 bc			611 ± 2 bc			600 ± 24 bc			411 ± 67 de			308 ± 58 e		
Nonanal	7 ± 0 ab			7 ± 1 ab			4 ± 0 c			6 ± 0 b			4 ± 1 c			6 ± 0 b			8 ± 1 a			4 ± 0 c			3 ± 0 c			4 ± 0 c			6 ± 1 b			4 ± 0 c			3 ± 0 c		
Benzaldehyde	11 ± 1 cdef			16 ± 3 b			5 ± 1 g			10 ± 1 cdef			13 ± 1 bcde			14 ± 1 bc			39 ± 3 a			7 ± 0 fg			9 ± 1 ef			10 ± 1 def			14 ± 1 bcd			10 ± 2 ef			14 ± 3 bc		
*Esters*																																							
Methyl acetate	18 ± 0 a			10 ± 1 de			14 ± 1 b			16 ± 0 a			13 ± 0 b			13 ± 1 b			10 ± 1 cd			16 ± 0 a			10 ± 1 cd			12 ± 0 bc			8 ± 0 e			9 ± 0 de			8 ± 0 e		
Ethyl acetate	10 ± 0 fg			12 ± 0 fg			5 ± 1 g			6 ± 0 g			5 ± 0 g			46 ± 2 c			144 ± 2 a			128 ± 19 b			37 ± 4 cd			31 ± 1 de			13 ± 1 fg			11 ± 1 fg			19 ± 2 ef		
Methyl butanoate	159 ± 5 c			173 ± 3 c			118 ± 15 d			118 ± 8 d			211 ± 3 b			226 ± 15 b			254 ± 31 a			87 ± 3 e			72 ± 11 e			91 ± 13 e			122 ± 4 d			168 ± 8 c			205 ± 5 b		
Ethyl butanoate	18 ± 2 cd			18 ± 1 cde			3 ± 0 e			5 ± 0 de			18 ± 1 cde			138 ± 10 a			138 ± 14 a			89 ± 9 b			13 ± 2 de			12 ± 2 de			30 ± 3 c			84 ± 10 b			135 ± 6 a		
Ethyl 3-methylbutanoate	1 ± 0 ef			1 ± 0 def			1 ± 0 def			1 ± 0 f			1 ± 0 ef			3 ± 1 b			3 ± 0 c			8 ± 0 a			1 ± 0 de			1 ± 0 ef			1 ± 0 ef			2 ± 0 d			3 ± 0 b		
Methyl hexanoate	330 ± 4 d			377 ± 24 c			140 ± 18 hi			149 ± 1 hi			306 ± 31 de			434 ± 19 b			534 ± 25 a			159 ± 16 gh			107 ± 13 i			128 ± 11 hi			195 ± 10 fg			216 ± 28 f			276 ± 5 e		
Butyl butanoate	19 ± 0 b			20 ± 3 b			18 ± 3 bc			18 ± 1 bc			17 ± 1 bcd			25 ± 3 a			20 ± 3 b			7 ± 1 f			11 ± 1 ef			13 ± 0 cde			12 ± 0 e			9 ± 1 ef			13 ± 2 de		
Ethyl hexanoate	21 ± 3 e			20 ± 2 ef			1 ± 0 g			3 ± 0 fg			15 ± 0 efg			162 ± 17 b			187 ± 16 a			71 ± 9 d			22 ± 3 e			26 ± 3 e			30 ± 2 e			91 ± 6 c			153 ± 1 b		
Hexyl acetate	10 ± 1 def			9 ± 0 ef			5 ± 1 f			7 ± 0 ef			29 ± 2 c			86 ± 11 a			82 ± 7 a			17 ± 3 de			9 ± 3 def			15 ± 0 def			14 ± 1 def			19 ± 3 d			48 ± 5 b		
*(2E)*-Hexenyl acetate	19 ± 2 c			18 ± 1 cd			9 ± 1 e			10 ± 1 e			20 ± 1 c			9 ± 1 e			15 ± 2 d			36 ± 4 a			17 ± 3 cd			31 ± 1 b			16 ± 1 cd			11 ± 1 e			9 ± 0 e		
Methyl octanoate	7 ± 1 cdef			7 ± 1 cdef			3 ± 0 hi			4 ± 0 efgh			8 ± 0 cd			18 ± 2 b			29 ± 4 a			4 ± 0 fgh			1 ± 0 i			4 ± 0 ghi			7 ± 0 cde			6 ± 2 defg			10 ± 1 c		
Hexyl butanoate	3 ± 0 c			2 ± 0 cd			2 ± 0 cd			1 ± 0 de			2 ± 0 cde			6 ± 1 b			11 ± 2 a			n.d.			n.d.			1 ± 0 cde			1 ± 0 cde			3 ± 0 c			5 ± 0 b		
Ethyl octanoate	n.d.			n.d.			n.d.			n.d.			n.d.			5 ± 1 b			6 ± 1 a			1 ± 0 d			n.d.			n.d.			1 ± 0 de			2 ± 0 c			4 ± 0 b		
*(2E)*-Hexenyl butanoate	1 ± 0 de			1 ± 0 e			1 ± 0 e			1 ± 0 de			3 ± 0 ab			2 ± 0 c			3 ± 0 ab			2 ± 0 cd			n.d.			n.d.			3 ± 0 ab			3 ± 1 a			3 ± 0 b		
2-Methyl butanoate	69 ± 12 d			59 ± 5 def			43 ± 3 efg			61 ± 3 de			72 ± 3 d			157 ± 4 b			238 ± 26 a			43 ± 3 efg			34 ± 0 g			40 ± 0 fg			38 ± 4 fg			67 ± 8 d			123 ± 4 c		
Benzyl acetate	546 ± 45 i			515 ± 22 i			819 ± 118 gh			3854 ± 46 a			2432 ± 44 d			3327 ± 102 b			3068 ± 134 c			655 ± 25 hi			970 ± 44 g			1990 ± 23 e			2411 ± 170 d			2160 ± 41 e			1420 ± 31 f		
Methyl 2-hydroxybenzoate	2 ± 0 de			2 ± 0 d			1 ± 0 gh			3 ± 0 a			2 ± 0 c			3 ± 0 b			3 ± 0 a			1 ± 0 h			1 ± 0 h			1 ± 0 g			1 ± 0 ef			1 ± 0 fg			1 ± 0 fg		
*Ketones*																																							
2-Butanone	16 ± 2 b			14 ± 2 b			13 ± 0 b			13 ± 1 b			9 ± 0 c			13 ± 1 bc			32 ± 5 a			n.d.			n.d.			n.d.			n.d.			n.d.			n.d.		
2,3-Butanedione	92 ± 4 a			87 ± 6 a			45 ± 8 c			46 ± 3 c			51 ± 3 bc			56 ± 4 b			42 ± 6 c			28 ± 0 de			25 ± 1 de			20 ± 2 e			32 ± 5 d			22 ± 4 de			25 ± 3 de		
2-Heptanone	10 ± 2 de			8 ± 0 e			2 ± 0 g			6 ± 0 f			12 ± 0 c			18 ± 0 a			20 ± 2 a			2 ± 0 g			2 ± 0 g			4 ± 0 fg			10 ± 1 de			12 ± 1 cd			15 ± 2 b		
*Alcohols*																																							
3,7-Dimethyl-1,6-octadien-3-ol	72 ± 5 d			76 ± 6 d			145 ± 11 bc			134 ± 5 bc			168 ± 1 a			138 ± 7 bc			81 ± 4 d			167 ± 6 a			144 ± 14 bc			127 ± 4 c			174 ± 10 a			152 ± 19 ab			138 ± 14 bc		
*(6Z)*-3,7,11-Trimethyl-1,6,10-dodecatrien-3-ol	191 ± 11 c			190 ± 14 c			142 ± 13 ef			156 ± 5 de			267 ± 5 a			240 ± 2 ab			227 ± 27 b			118 ± 15 f			132 ± 3 ef			146 ± 2 ef			237 ± 31 ab			183 ± 14 cd			207 ± 12 bc		
*Acids*																																							
Butyric acid	2 ± 0 de			1 ± 0 e			1 ± 0 e			2 ± 0 de			3 ± 0 de			8 ± 0 bc			15 ± 3 a			1 ± 0 e			1 ± 0 e			1 ± 0 e			3 ± 0 d			7 ± 0 c			9 ± 0 b		
Hexanoic acid	87 ± 7 fg			96 ± 9 fg			62 ± 7 h			102 ± 6 f			185 ± 17 e			381 ± 10 c			664 ± 21 a			59 ± 1 h			71 ± 6 gh			112 ± 12 f			231 ± 7 d			380 ± 12 c			502 ± 13 b		
*Furanones*																																							
4-Methoxy-2,5-dimethyl-3(2*H*)-furanone	156 ± 14 fg			167 ± 6 fg			101 ± 7 g			229 ± 10 f			376 ± 39 e			815 ± 8 c			1137 ± 66 a			156 ± 4 fg			112 ± 2 g			210 ± 9 f			338 ± 21 e			571 ± 18 d			985 ± 87 b		
Sum	3787 ± 5 g			3836 ± 102 g			3490 ± 219 g			6651 ± 61 c			5766 ± 17 d			7742 ± 256 b			8186 ± 286 a			2968 ± 68 h			3048 ± 136 h			4200 ± 64 f			5129 ± 51 e			5110 ± 228 e			5130 ± 272 e		

Different letters within the same row denote significant differences in the same compound (*p* < 0.05).

## Supplementary Materials

The following supporting information can be downloaded at: https://www.mdpi.com/article/10.3390/molecules27196599/s1, Table S1: Multivariate analysis of variance F-values and p-values for the volatile contents of all strawberries.
