# Supplementary material for: Changes in Volatile Compounds in Short-Term High CO2-Treated ‘Seolhyang’ Strawberry (Fragaria × ananassa) Fruit during Cold Storage
Source: Molecules. 2022 Oct 5;27(19):6599. doi: 10.3390/molecules27196599 (PMC9571338; doi:10.3390/molecules27196599)
Supplement: Supplementary file 1 [file molecules-27-06599-s001.zip › molecules-1897139-supplementary.pdf]

Table S1. Multivariate analysis of variance F-values and *p*-values for the volatile contents of all strawberry samples.

Compound code	1	9	12	18	22	2	3	6	7	8	11
F-value											
maturity	3.4	17.9	36.5	0.6	79.6	88.5	309.4	629.9	524.8	198.5	1376.5
CO_2	1739.3	16.3	43.0	8.4	619.8	147.0	4.7	524.0	34.6	0.4	575.4
time	6.8	143.8	128.4	9.0	512.3	18.8	245.3	156.8	545.0	126.1	138.3
maturity * CO_2	0.9	2.1	0.0	84.2	118.8	14.3	27.2	0.1	114.5	173.8	107.7
maturity * time	0.8	9.2	3.3	18.4	106.3	35.7	39.5	24.4	66.8	62.0	92.0
CO_2 * time	2.7	5.1	11.9	42.8	393.5	57.6	232.0	6.4	115.1	148.0	25.9
maturity * CO_2 * time	4.8	0.9	3.3	11.5	110.4	12.5	177.7	22.8	54.0	67.1	35.5
p-value											
maturity	NS	***	***	NS	***	***	***	***	***	***	***
CO_2	***	***	***	**	***	***	*	***	***	NS	***
time	***	***	***	***	***	***	***	***	***	***	***
maturity * CO_2	NS	*	NS	***	***	***	***	NS	***	***	***
maturity * time	NS	***	*	***	***	***	***	***	***	***	***
CO_2 * time	*	**	***	***	***	***	***	***	***	***	***
maturity * CO_2 * time	**	NS	*	***	***	***	***	***	***	***	***

Table S1. (Continued)

Compound code	13	14	15	16	17	19	20	21	26	27	28
F-value											
maturity	21.6	1455.8	33.9	293.1	365.9	9.9	408.7	66.5	254.5	383.4	85.3
CO_2	190.2	1.0	485.0	16.1	247.4	156.2	10.4	483.3	475.9	1314.0	268.7
time	1.3	327.7	345.9	122.9	138.8	103.1	127.4	579.4	315.3	815.3	35.6
maturity * CO_2	18.7	0.1	23.5	194.4	49.9	8.9	10.4	421.5	4.9	286.6	15.5
maturity * time	2.7	239.5	38.8	82.1	38.7	7.5	127.4	372.8	19.9	131.8	7.3
CO_2 * time	2.9	34.9	171.2	47.9	42.3	67.5	16.4	530.8	75.9	99.6	20.9
maturity * CO_2 * time	9.0	41.6	77.1	85.3	27.6	24.2	16.4	476.2	16.7	34.9	10.5
p-value											
maturity	***	***	***	***	***	**	***	***	***	***	***
CO_2	***	NS	***	***	***	***	**	***	***	***	***
time	*	***	***	***	***	***	***	***	***	***	***
maturity * CO_2	***	NS	***	***	***	**	**	***	*	***	***
maturity * time	*	***	***	***	***	***	***	***	***	***	***
CO_2 * time	*	***	***	***	***	***	***	***	***	***	***
maturity * CO_2 * time	***	***	***	***	***	***	***	***	***	***	***

Table S1. (Continued)

Compound code	4	5	10	23	30	25	29	24	Sum of volatile content
F-value									
maturity	223.7	485.6	955.1	84.0	1420.7	305.7	3081.3	874.7	0.6
CO_2	852.1	583.4	191.9	15.4	47.1	20.0	243.5	198.2	2069.7
time	67.5	38.5	164.5	55.4	59.4	89.1	2068.8	944.8	706.5
maturity * CO_2	24.6	44.9	7.7	68.0	10.6	6.6	43.2	15.3	175.8
maturity * time	7.9	33.1	87.3	21.0	29.0	90.9	370.5	65.7	39.0
CO_2 * time	104.5	11.9	14.9	23.7	2.9	4.1	150.4	64.6	91.3
maturity * CO_2 * time	10.3	19.4	9.8	6.5	5.2	10.2	7.3	6.6	21.5
p-value									
maturity	***	***	***	***	***	***	***	***	NS
CO_2	***	***	***	***	***	***	***	***	***
time	***	***	***	***	***	***	***	***	***
maturity * CO_2	***	***	**	***	**	*	***	***	***
maturity * time	***	***	***	***	***	***	***	***	***
CO_2 * time	***	***	***	***	*	**	***	***	***
maturity * CO_2 * time	***	***	***	**	**	***	***	***	***

Number corresponded with peak no. in Table 1

NS stands for not significant ($p > 0.05$).

*, **, and *** indicate significantly different at $p < 0.05$, $p < 0.01$, and $p < 0.001$, respectively.
